# Identification of the microbial diversity after fecal microbiota transplantation therapy for chronic intractable constipation using 16s rRNA amplicon sequencing

**DOI:** 10.1371/journal.pone.0214085

**Published:** 2019-03-19

**Authors:** Tadashi Ohara

**Affiliations:** Department of Intestinal Biosciences and Medicine, Fukushima Medical University, Fukushima, Japan; National Institute for Agronomic Research, FRANCE

## Abstract

**Background:**

Fecal microbiota transplantation (FMT) is an effective therapeutic approach for the treatment of functional gastrointestinal disease by restoring gut microbiota; however, there is a lack of sufficient understanding regarding which microbial populations successfully colonize the recipient gut. This study characterized microbial composition and diversity in patients diagnosed with chronic constipation at 1 month and 1 year after FMT.

**Methods:**

We explored the microbial diversity of pre- and posttransplant stool specimens from patients using 16S rRNA gene sequencing, followed by functional analysis.

**Results:**

The results identified 22 species of microorganisms colonized in the recipients from the donors at 1 month after FMT. One-year follow-up of the patient identified the colonization of 18 species of microorganisms, resulting in identification of species in significant abundance, including *Bacteroides fragilis* and *Hungatella hathewayi* in the recipient at 1 month after FMT and *Dialister succinatiphilus*, *Coprococcus catus*, and *Sutterella stercoricanis* at 1 year after FMT. The majority of the colonized species belong to the phylum *Firmicutes* and carry genes related to polysaccharide metabolism and that enhance the energy-harvesting efficiency of the host.

**Conclusion:**

These results suggest that FMT is effective for the treatment of chronic constipation through the restoration and colonization of donor microbiota in the recipient gut up to 1 year after FMT.

## Introduction

Chronic constipation is a functional gastrointestinal disorder that has a global prevalence rate of 8.2% to 32.9% [1]. Although there is no specific prevalence rate available for Japan, the incidence rate varies from 8.2% to 16.8% in Asian countries [1]. Alterations of intestinal microbiota are closely related to constipation and constipation-related symptoms [2], and the diversity of intestinal microflora plays a role in the ability of the host to absorb nutrients and regulate immunological responses [3]. Common treatment strategies for chronic constipation include laxatives, dietary changes, lifestyle changes, and improvement of gut microflora through probiotics, prebiotics, synbiotics, and fecal microbiota transplantation (FMT). Due to its safety, convenience, and efficacy, FMT is now a widely accepted treatment for *Clostridium difficile* infection (CDI) and also promising for the treatment of chronic constipation [4,5].

FMT involves the administration of liquid filtrate of feces from a healthy donor into the gut of a recipient in order to cure a specific disease. Administration of the fecal suspension is performed through a nasogastric or nasoduodenal tube, colonoscope, enema, or capsule. The donor stool is sourced from the next of kin of the recipient or a healthy donor and tested for any pathological organisms before transplantation [6,7]. FMT has been routinely used for the treatment of chronic constipation with clinical success, with improvement rates ranging from 87% to 90% and 67.4% to 76.2%, respectively [8–10]. Additionally, the route of administration plays a role in FMT outcome and efficacy, as a systematic report showed that administration through colonoscopy and enema resulted in a higher success rate as compared with administration through gastroscopy and the nasogastric mode of delivery for CDI treatment [11]. However, there are no studies directly comparing FMT-delivery routes in chronic constipation. The transplantation of fecal microbiota from a donor results in the engraftment of new microbial species, as well as augmentation of existing species, in the recipient. It is understood that the fecal microbiota in the recipient changes substantially, with microbial diversity becoming similar to that of the donor microbiota [5,12–15]. Therefore, the restoration of microbial diversity determines the success of FMT; however, the donor microbial profile and selection strategy are equally important determinants for its success [16].

Successful restoration of microbial diversity is usually determined using high-throughput sequencing technologies to investigate the ecology of the microbial population. Several studies report using 16s rRNA amplicon sequencing, shotgun metagenomics, and metagenomic assembly and binning to investigate microbial diversity following FMT [17–20]. However, few studies have investigated the efficacy of FMT for colonizing the microbial population over time. Because gut microbiota is highly dynamic and constantly influenced by dietary habits, it is critical to analyze FMT effectiveness at restoring and colonizing microbial diversity over time. We previously reported the use of FMT in a clinical case of chronic intractable constipation, finding that FMT resulted in the long-term incorporation of microbes, especially *Clostridium* and *Bifidobacterium*, in recipient intestinal flora [21]. That study was a preliminary analysis using terminal fragment-length polymorphism analysis following DNA extraction from the feces. There have been no subsequent reports of FMT use to treat chronic intractable constipation; therefore, we performed 16s rRNA amplicon sequencing on the same donor and recipient samples in order to analyze FMT efficacy at 1 month and 1 year after transplantation.

## Materials and methods

### Ethics statement

Our study protocol was reviewed and approved by the Fukushima Daiichi Hospital Institutional Ethics Review Committee of Fukushima Medical University (Fukushima, Japan). FMT treatment was performed at Fukushima Daiichi Hospital, and all experiments were performed in accordance with the 1964 Declaration of Helsinki and its later amendments. Patients and donors of fecal material were informed of the potential risks and benefits of fecal transplantation and its experimental nature. Patients provided written, informed consent before participating.

### Sample collection and DNA extraction

Feces from donors and recipients (before and at 1 month and 1 year after FMT) were immediately suspended in a solution containing 100 mM Tris-HCl (pH 8.0), 40 mM Tris-EDTA (pH 8.0), 4 M guanidine thiocyanate, and 0.0001% bromothymol blue. An aliquot of 1200 μL of the suspension was homogenized with zirconia beads in a 2.0-mL screw-cap tube in a FastPrep 24 instrument (MP Biomedicals, Illkirch, France) at 5 m/s for 2 min and placed on ice for 1 min. After centrifugation at 5000 rpm for 1 min, DNA was extracted from 200 μL of the suspension using an automatic nucleic acid extractor (Precision System Science, Chiba, Japan). MagDEA DNA 200 (GC) (Precision System Science) was used as the reagent for automatic nucleic acid extraction [22, 23].

### 16S rRNA gene amplification and sequencing

Sequences of the 16S rRNA gene of donor and recipient fecal microbiota were analyzed by next-generation sequencing using the MiSeq system (Illumina, San Diego, CA, USA), as previously described [24]. The V3 and V4 hypervariable regions of the 16S rRNA gene were amplified by polymerase chain reaction (PCR) from microbial genomic DNA using prokaryote universal primer sets: forward primer, 5′-AATGATACGGCGACCACCGAGATCTACACXXXXXXXXACACTCTTTCCCTACACGACGCTCTTCCGATCTCCTACGGGNBGCASCAG-3′ [Xs represent the sample-specific 8-bp barcode sequences, (TAAGGCGA)] and reverse primer, 5′-CAAGCAGAAGACGGCATACGAGATZZZZZZZZGTGACTGGAGTTCAGACGTGTGCTCTTCCGATCTGACTACNVGGGTATCTAATCC-3’ [Zs represent the sample-specific 8-bp barcode sequences (CTCTCTAT, TATCCTCT, GTAAGGAG, and ACTGCATA]. The underlined sequences represent the PCR primer region (Pro341F and Pro805R) used for the dual-index method [25]. The V3 and V4 regions of the 16S rRNA fragments were amplified in 25-μL PCR reactions containing ~30 ng DNA template, 12.5 μL 2× MightyAmp Buffer (v.2.0; Mg^2+^, dNTP plus; TaKaRa Bio, Inc., Shiga, Japan), 0.25 μM of each primer, and 0.625 U MightyAmp DNA polymerase (TaKaRa Bio, Inc.) [23]. The PCR conditions for DNA amplification were as follows: initial denaturation at 98°C for 2 min, followed by 35 cycles of annealing beginning at 65°C and ending at 55°C for 15 s and extension at 68°C for 30 s. The annealing temperature was lowered 1°C every cycle until it reached 55°C, which was maintained for the remaining cycles. PCR products were purified through a MultiScreen PCR_u96_ filter plate (Merck Millipore, Billerica, MA, USA). To prepare the amplicon pool, purified products were quantified by real-time quantitative PCR on a Rotor-Gene Q quantitative thermal cycler using MightyAmp reagents and SYBR Plus (TaKaRa Bio, Inc.) [24]. Barcoded amplicons were sequenced using paired ends and the MiSeq reagent kit (v.3.0; Illumina) on the MiSeq system (600 cycles, 2× 284 bp/cycle; Illumina) [26].

### Read alignment and quality check

Paired-end reads were concatenated using the FASTQ-join program with default options (Erik A. 2011). Only joined reads with quality value score ≥20 for >99% of the sequence were extracted using the FASTX-Toolkit (http://hannonlab.cshl.edu/fastx_toolkit/). Chimeric sequences were deleted using uSearch version 6.1 software [27].

### 16S rRNA sequence data analysis

Bacterial identification from analyses of the sequence reads was performed using Metagenome@KIN software (v.2.2.1; World Fusion, Tokyo, Japan) and the TechnoSuruga Lab Microbial Identification database (DB-BA 10.0; TechnoSuruga Laboratory, Shizuoka, Japan) using a homology threshold of ≥97%.

### Analysis of microbial-compositional structure

To indicate specific or common taxonomies among the microbial-compositional structures of the donor and recipient samples, Venn diagrams were constructed using the R Venn Diagram package (https://www.r-project.org/) based on the microbial-community results returned from DB-BA 10.0 analysis [25]. Heatmap clustering of the bacterial taxonomies identified in the fecal microbiota by the R amap and gplots packages was performed to visualize the abundance of taxonomies and similar microbial-compositional structures [28]. Data for each sample were aligned to a dendrogram constructed using the Ward algorithm based on a Euclidean distance matrix.

### Functional analysis of recipient gut microbiota and microbial-compositional structure

Operational taxonomic units (OTUs) were constructed using the raw data reads. To join two paired-end reads, we used FASTQ-join software with default options [29], and chimeric sequences were deleted with uSearch version 6.1 software [30]. OTUs at 97% sequence similarity were selected using the Greengenes database (http://greengenes.secondgenome.com/) and pick_open_reference_otus.py in the QIIME 1.8.0 pipeline [29]. Functional analysis of donor and recipient microbiota based on the Kyoto Encyclopedia of Genes and Genomes Orthology (KO) database was performed using PICRUSt version 1.1.1 [31].

### Statistical analysis

Samples were allocated into donor and recipient groups. Continuous data were presented as the mean±standard deviation, whereas categorical data were presented as numbers (%). Statistical analysis was performed with repeated measures analysis of variance for continuous variables and Pearson’s chi-squared test for categorical variables. Differences in taxa abundance between groups were measured using an unpaired *t* test (assuming unequal variance), with significance determined at *p* < 0.05. All data were analyzed using SPSS (v.20.0; IBM Corp. Armonk, NY, USA).

## Results

### Analysis of the 16S rRNA sequences of donor and recipient samples

A total of four samples (one each from the donor and recipient before FMT, one recipient sample at 1 month after FMT, and one recipient sample at 12 months after FMT) were subjected to 16S rRNA sequencing. We obtained an average sequence depth of 95,464 reads per sample (range: 58,972–172,877), and after quality filtering, the sample depth across all samples was normalized to 49,243 reads per sample and used for analysis. We identified a total of 469 OTUs.

**Fig 1:**
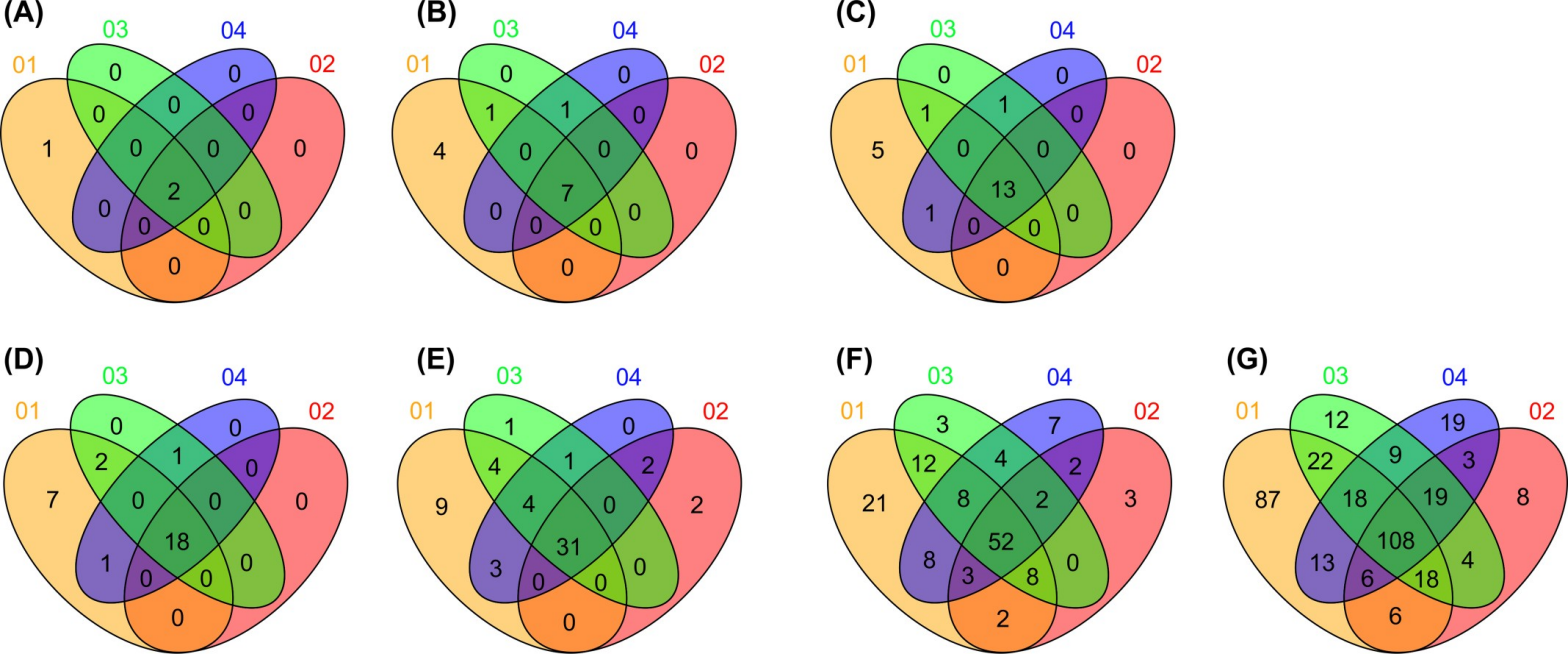
Venn diagram Showing Time-dependent Taxonomy Classification (from Kingdom to Species) of the Microbial-compositional Structures for Donor and Recipient Samples. The Venn diagram shows the number of common microbial taxonomies according to overlapping regions. Sample identifiers: 01 corresponds to the donor, 02 to the recipient (pre-FMT), 03 to the recipient (at 1 month after FMT), and 04 to the recipient (at 1 year after FMT). Microbial-compositional structure is presented according to (A) kingdom (B) phylum, class, (D) order, (E) family, (F) genus, and (G) species.

**Fig 2:**
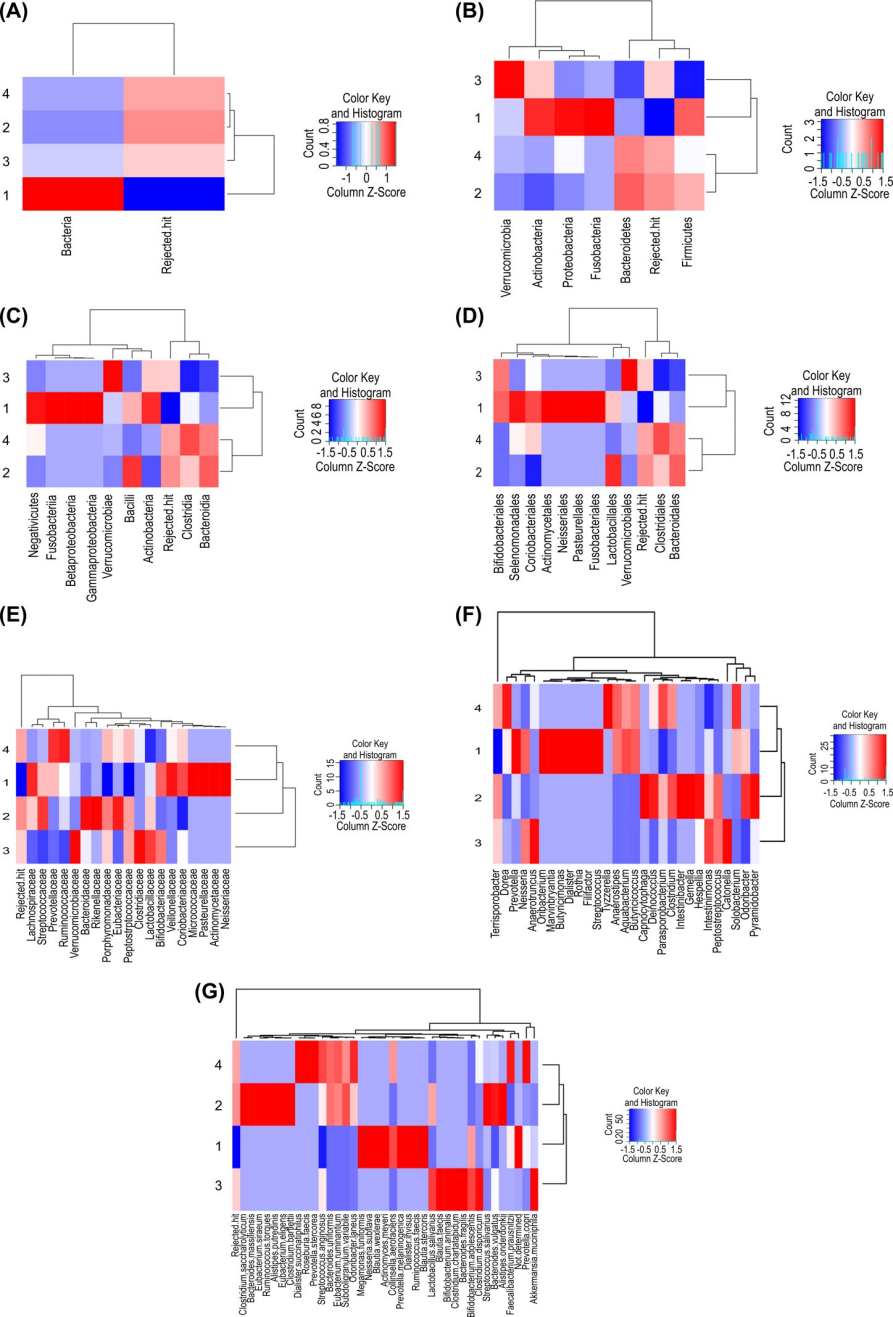
Clustering of Fecal Microbiota by Taxonomy (from Kingdom to Species). The color scale of the heatmap shows the abundance of each bacterial taxonomy according to Z score. Data are presented according to (A) kingdom, (B) phylum, (C) class, (D) order, (E) family, (F) genus, and (G) species.

From these sequence reads, a range of 72 to 154 genera was identified in stool specimens. The average number of genera was 123 for the donors, 96 for the recipients pre-FMT, and 120 for the recipients post-FMT. Two failed FMTs resulted in recipients with an average number of genera of 90. Therefore, clinically successful FMT resulted in an average increase in genera of 25% for recipients post-FMT as compared with pre-FMT (extraction of differential-gene-expression analysis; *p* < 0.05). We identified 90 species belonging to genera showing significant changes in post-FT abundance. Donor samples returning successful sequence classification contained a median of 211 unique species, whereas classifications for pre- and post-FMT samples consisted of a median of 164 and 255 unique species, respectively. The microbial-compositional structures of the donors and recipients are presented in Fig 1, and data for each sample were aligned to a dendrogram constructed using the Ward algorithm based on Euclidean distance (Fig 2).

### Species-level changes following FMT

**Fig 3:**
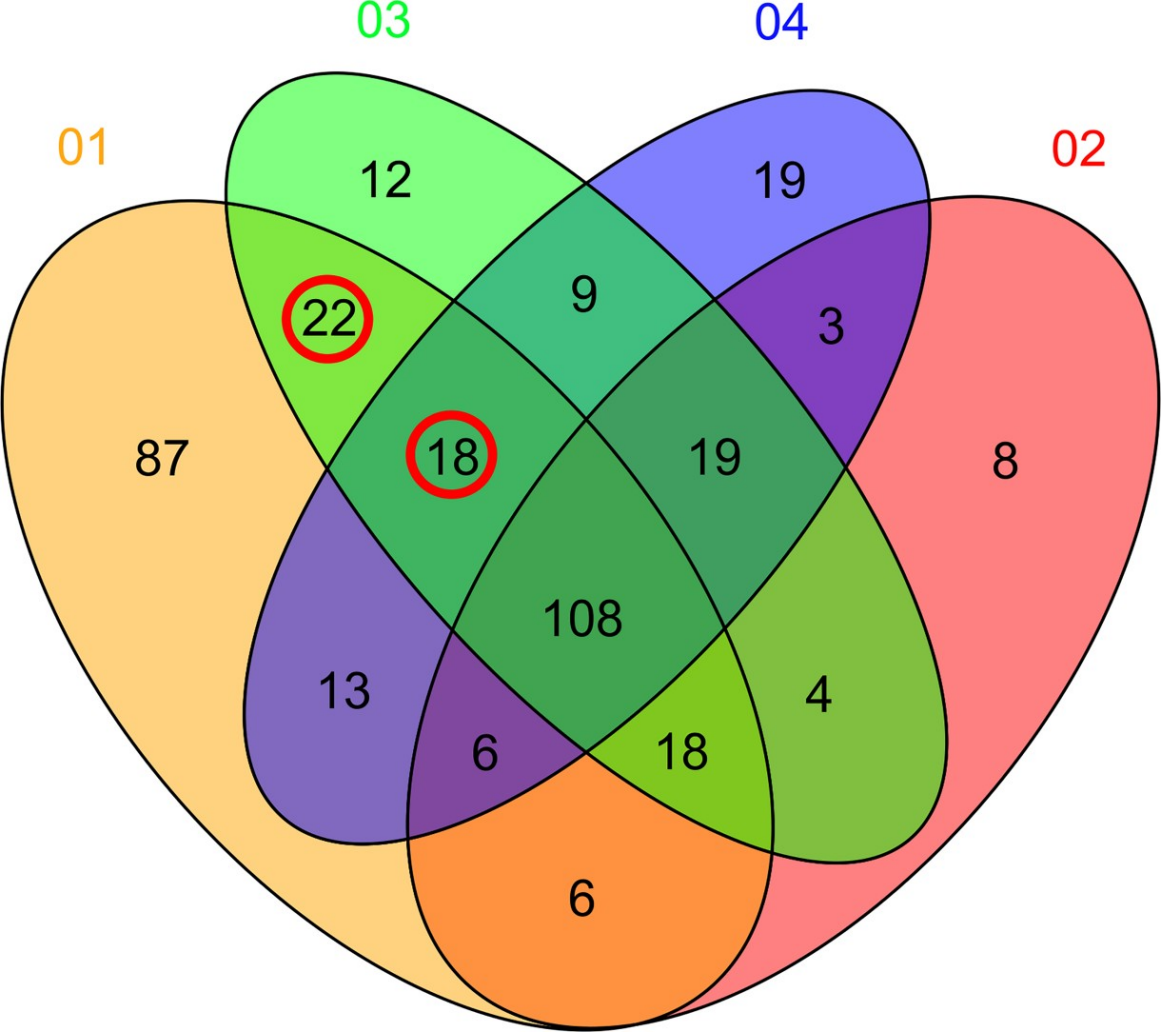
Sequence Similarity of Microorganisms Colonized in Recipients at 1 Month and 1 Year after FMT.

At 1 month after FMT, we observed a general shift in recipient fecal microbiota composition toward the donor microbial composition. Examination of changes in the microbiota at the species level following FMT showed two trends. At 1 month after FMT, analysis of the microbiota in the recipient identified 22 species of donor microorganisms (Fig 3 and Table 1), whereas 18 species of donor microorganisms were identified in the recipient at 1 year after FMT (Fig 3 and Table 2). Among the identified species, we found a significant abundance of *Bacteroides fragilis* and *Hungatella hathewayi* in recipients at 1 month after FMT and of *Dialister succinatiphilus*, *Coprococcus catus*, and *Sutterella stercoricanis* at 1 year after FMT.

**Table 1:** Microorganisms Colonized in Recipients at 1 Month after FMT.

No.	Donor	Recipient	One-month post-FMT	One-year post-FMT	Species
1	2	0	1	0	*Acinetobacter lwoffii*
2	48	0	2	0	*Actinomyces graevenitzii*
3	34	0	1	0	*Bacteroides dorei*
4	4	0	453	0	*Bacteroides fragilis*
5	26	0	1	0	*Catonella morbi*
6	1	0	1	0	*Clostridium indolis*
7	1	0	18	0	*Clostridium innocuum*
8	3	0	1	0	*Clostridium lactatifermentans*
9	14	0	6	0	*Clostridium symbiosum*
10	3	0	3	0	*Eubacterium desmolans*
11	1	0	1	0	*Gemella morbillorum*
12	5	0	2	0	*Gordonibacter pamelaeae*
13	1	0	209	0	*Hungatella hathewayi*
14	57	0	3	0	*Lactococcus lactis*
15	3	0	3	0	*Mucispirillum schaedleri*
16	11	0	2	0	*Peptostreptococcus stomatis*
17	9	0	1	0	*Prevotella oris*
18	33	0	1	0	*Rothia nasimurium*
19	15	0	1	0	*Solobacterium moorei*
20	20	0	1	0	*Stomatobaculum longum*
21	11	0	1	0	*Streptococcus lactarius*
22	7	0	1	0	*Veillonella rogosae*

**Table 2:** Microorganisms Colonized in recipients at 1 year after FMT.

No.	Donor	Recipient	One-month post-FMT	One-year post-FMT	Species
1	2	0	6	2	*Anaerotruncus colihominis*
2	16	0	3	3	*Asaccharobacter celatus*
3	48	0	15	2	*Bifidobacterium pseudolongum*
4	2	0	29	1	*Clostridium bolteae*
5	2	0	64	9	*Coprobacter fastidiosus*
6	16	0	33	112	*Coprococcus catus*
7	1	0	64	14	*Desulfovibrio desulfuricans*
8	3	0	65	320	*Dialister succinatiphilus*
9	3	0	36	2	*Eubacterium callanderi*
10	4	0	9	2	*Flavonifractor plautii*
11	74	0	2	3	*Gemella sanguinis*
12	2	0	4	2	*Intestinimonas butyriciproducens*
13	103	0	11	4	*Leptotrichia wadeii*
14	43	0	1	2	*Megasphaera micronuciformis*
15	13	0	1	1	*Prevotella oulorum*
16	14	0	13	92	*Sutterella stercoricanis*
17	6	0	1	1	*Veillonella ratti*
18	9	0	1	13	*Veillonella tobetsuensis*

### Principal coordinate analysis (PCA)

**Fig 4:**
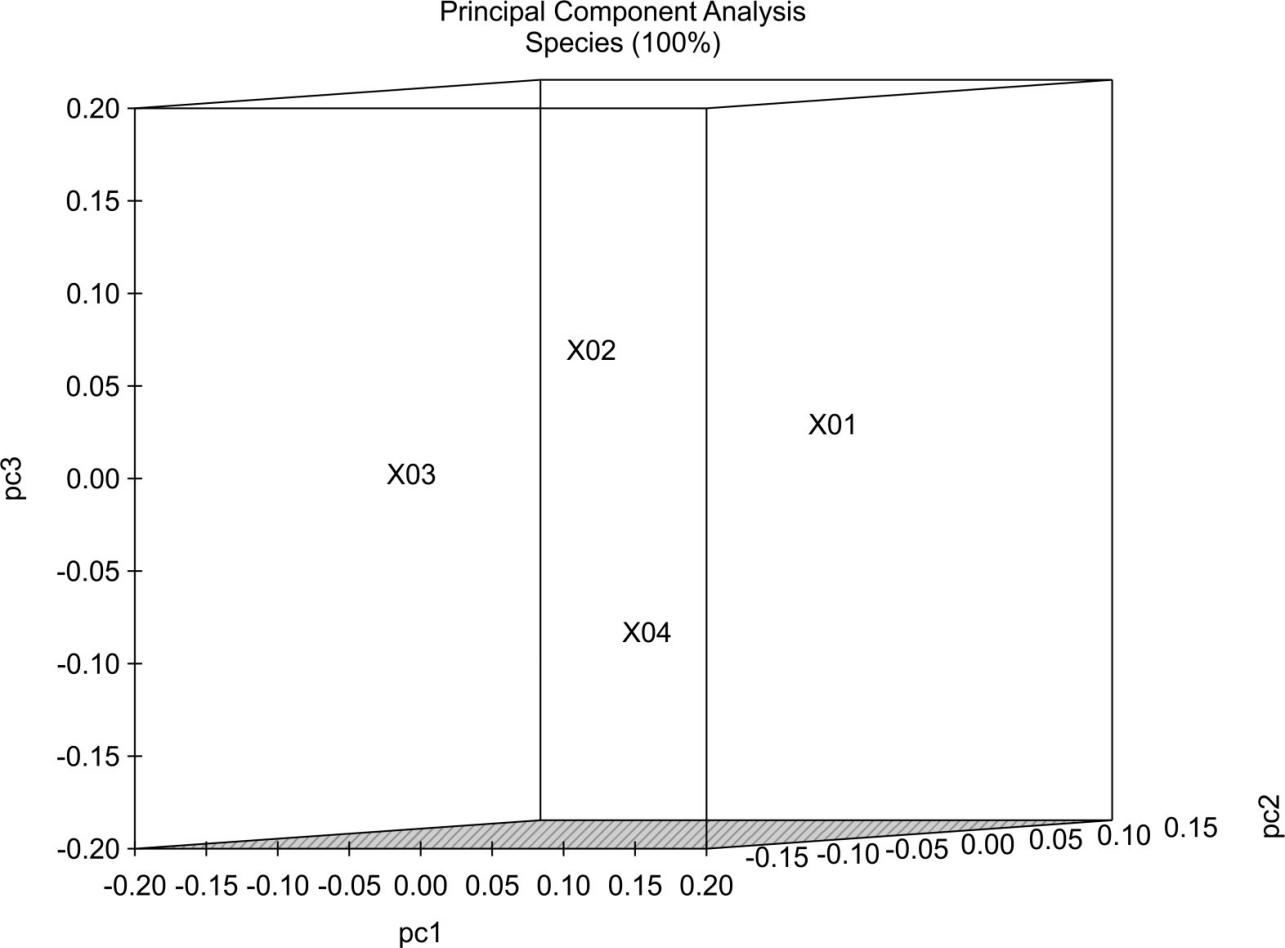
PCA of community similarity according to phylogenetic branch length (UniFrac) in the 16S rRNA sequences.

We used PCA to analyze OTU variability between donor samples and those from recipients at 1-month and 1-year post-FMT samples. The first three axes of PCA results plotted in Fig 4 accounted for 79.3% of the total variability at the species level. Notably, the variability was higher than the inter-individual variability between samples. The relative contribution to variability for each successive axis decreased, with the first five axes accounting for all non-random variability. It is possible that clustering might have been responsive to both differences in proportions and the presence or absence of sequence data.

### Functional analysis

Functional analysis of donor and recipient microbiota based on the KO database identified sialidase-1 [EC:3.2.1.18] (K01186). We found no significant difference in propionic acid and butyric acid systems or the number of genes related to the mucin-degrading system between donor and recipient samples after FMT.

## Discussion

This was a prospective study with a long-term follow-up performed to comprehensively identify microbiota-colonization potential following FMT in patients with chronic constipation. To the best of our knowledge, this is the first study characterizing differences in gut microbiota at 1 month and 1 year after FMT. We identified 22 microorganisms at 1-month post-FMT and 18 at 1-year post-FMT that had colonized in recipients from donors. This result indicates that large-scale metagenomic surveys might be useful for rapid prediction of the colonization properties of bacterial populations and provides insight into possible future directions for investigating the ecological and functional bases of gut colonization.

One of the early events indicating successful transplantation is the ability of the transplanted microbiota to access gastrointestinal niches [23]. Once donor microbes reach these areas, they compete for nutrients and space to expand their number. To gain insight into the reconstitution of the microbial community in FMT patients, we identified the number and type of species colonized in the recipients at 1 month and 1 year after FMT. Although the recipients harbored 18 species of donor microorganisms colonized after transplantation, it is important to note that recipient eating habits did not change significantly after FMT. We observed a significant abundance of *B*. *fragilis* and *H*. *hathewayi* in recipients at 1 month after FMT and *D*. *succinatiphilus*, *C*. *catus*, and *S*. *stercoricanis* at 1 year after FMT.

Gut microbiota is involved in the fermentation of indigestible polysaccharides (components of dietary fibers) that are subsequently converted into short-chain fatty acids (SCFAs; e.g., acetate, propionate, and butyrate) [32] used by the host as an energy source and representing 10% to 15% of the energy influx from food [33]. The five species identified in recipients at 1-month and 1-year post-FMT belong to the phylum *Firmicutes* and carry genes related to polysaccharide metabolism and involved in enhancing efficient energy harvesting by the host. Parthasarathy et al. [34] recently reported that analysis of colonic mucosa-associated microbiota successfully discriminated 25 adult patients with constipation from 25 healthy adults with 94% accuracy. Consistent with this finding report, our analysis in the present study identified a greater abundance of genera within the phylum *Bacteriodetes*. In previous phylogenetic analyses, *H*. *hathewayi* was closely related to *Clostridium hathewayi* DSM 13479(T) (97.84% similarity), a member of rRNA gene-cluster XIVa of the genus *Clostridium*, and formed a coherent cluster with other related members of the *Blautia* (*Clostridium*) *coccoides* rRNA group [35]. This species is responsible for the production of acetate and propionate, which might be involved in gastrointestinal mobility. Similarly, *D*. *succinatiphilus* and *C*. *catus* were also reported as involved in SCFA production, where they decarboxylate succinate to propionate [36]. Interestingly, we also identified an abundance of *S*. *stercoricanis*, previously associated with better outcomes in patients with inflammatory bowel disease, as well as with resistance to the development of experimental autoimmune encephalomyelitis tumor necrosis factor receptor-2-knockout mice [37–39].

Although previous studies report successful medical outcomes associated with FMT [8, 40, 41], a thorough understanding of this procedure from the perspective of microbial ecology is still lacking. Investigation of FMT as an ecological event and identification of the key components that facilitate procedure success as a treatment for intestinal disorders requires detailed characterization of the transferred microbial populations at an appropriate level of resolution.

Our study has several limitations that might have resulted in decreased confidence in inferring the metagenomic results. First, changes in the diversity of fecal microbiota could have occurred as a result of dietary and lifestyle-related changes and might not be attributable to FMT. Additionally, there are limitations inherent to the employed methodology. For example, 16S rRNA gene sequencing does not sample antibiotic resistance genes and is therefore, unable to determine whether organisms detected in a clinical sample possess such genes. Second, 16S rRNA gene sequencing is limited in resolution, as it only distinguishes bacteria to the genus level. To overcome this limitation and obtain resolution at the species level, we performed a homology search. However, clear separation of the pre-FMT/failed-FMT and donor/successful-FMT groups suggested that the presence or absence of key taxa likely outweighed inter-individual differences in the determination of FMT success. Although variability in microbiota composition is dependent upon historical exposure to microbes and antibiotics, the post-FMT samples grouped closely with donor samples associated with successful FMT, suggesting the presence of key genera required for recolonization and bacterial-community resilience.

In conclusion, our analyses indicated that FMT resulted in effective colonization of donor microbiota in the recipient gut up to 1 year after FMT. The clinical implications of our findings for the development of a viable probiotic drug to treat chronic constipation are significant. It is conceivable that a probiotic containing the species identified in this study as significantly increased in recipients following FMT might be sufficient to restore gut homeostasis. Larger randomized, controlled trials are needed to further assess the benefits and risks of FMT for constipation.
